# Macrophage subpopulations in pediatric patients with lupus nephritis and other inflammatory diseases affecting the kidney

**DOI:** 10.1186/s13075-024-03281-1

**Published:** 2024-02-08

**Authors:** Mira Sandersfeld, Maike Büttner-Herold, Fulvia Ferrazzi, Kerstin Amann, Kerstin Benz, Christoph Daniel

**Affiliations:** 1 Department of Nephropathology, Institute of Pathology, Friedrich-Alexander-University Erlangen-Nürnberg (FAU), Krankenhausstr. 8-10, Erlangen, 91054 Germany; 2 Institute of Pathology, FAU Erlangen-Nürnberg, Erlangen, 91054 Germany; 3 Department of Pediatrics, FAU Erlangen-Nürnberg, Erlangen, 91054 Germany

**Keywords:** Pediatric patients, Inflammatory kidney diseases, Lupus nephritis, Macrophages, Macrophage subtypes

## Abstract

**Background:**

Macrophages play an important role in the pathogenesis of lupus nephritis (LN), but less is known about macrophage subtypes in pediatric LN. Here we compared renal inflammation in LN with other inflammatory pediatric kidney diseases and assessed whether inflammation correlates with clinical parameters.

**Methods:**

Using immunofluorescence microscopy, we analyzed renal biopsies from 20 pediatric patients with lupus nephritis (ISN/RPS classes II–V) and pediatric controls with other inflammatory kidney diseases for infiltration with M1-like (CD68 + /CD206 − , CD68 + /CD163 −), M2a-like (CD206 + /CD68 +), and M2c-like macrophages (CD163 + /CD68 +) as well as CD3 + T-cells, CD20 + B-cells, and MPO + neutrophilic granulocytes. In addition, the correlation of macrophage infiltration with clinical parameters at the time of renal biopsy, e.g., eGFR and serum urea, was investigated. Macrophage subpopulations were compared with data from a former study of adult LN patients.

**Results:**

The frequency of different macrophage subtypes in biopsies of pediatric LN was dependent on ISN/RPS class and showed the most pronounced M1-like macrophage infiltration in patients with LN class IV, whereas M2c-like macrophages were most abundant in class III and IV. Interestingly, on average, only half as many macrophages were found in renal biopsies of pediatric LN compared to adult patients with LN. The distribution of frequencies of macrophage subpopulations, however, was different for CD68 + CD206 + (M2a-like) but comparable for CD68 + CD163 − (M1-like) CD68 + CD163 + (M2c-like) cells in pediatric and adult patients. Compared to other inflammatory kidney diseases in children, fewer macrophages and other inflammatory cells were found in kidney biopsies of LN. Depending on the disease, the frequency of individual immune cell types varied, but we were unable to confirm disease-specific inflammatory signatures in our study due to the small number of pediatric cases. Worsened renal function, measured as elevated serum urea and decreased eGFR, correlated particularly strongly with the number of CD68 + /CD163 − M1-like macrophages and CD20 + B cells in pediatric inflammatory kidney disease.

**Conclusion:**

Although M1-like macrophages play a greater role in pediatric LN patients than in adult LN patients, M2-like macrophages appear to be key players and are more abundant in other pediatric inflammatory kidney diseases compared to LN.

**Supplementary Information:**

The online version contains supplementary material available at 10.1186/s13075-024-03281-1.

## Introduction

Systemic lupus erythematosus (SLE) is an autoimmune disease with a heterogeneous clinical picture. Lupus nephritis (LN) is manifested in 50–80% of all SLE patients, with varying degrees of severity [1]. The International Society of Nephrology/Renal Pathology Society (ISN/RPS) classification characterized LN classes in 2003 and revised them in 2018. It divides the pathology into 6 classes according to histological findings. Class I, minimal mesangial LN; class II, mesangial proliferative LN; class III, focal LN; class IV: diffuse LN; class V, membranous LN, and class VI, advanced sclerosing LN (very rarely seen) [2]. Although lupus nephritis is an autoimmune disease with so far unclear presumably multifactorial causes and therefore the same histological changes do not necessarily have the same trigger [3, 4], there are some characteristics that determine the clinical picture in all patients [5]. Antibodies directed against double-stranded DNA have been shown to play a crucial role in the pathogenesis of SLE [3, 4, 6]. Deposits of immune complexes in the glomeruli, which are accompanied by complement activation, are also of great importance for renal pathology [7–10]. In addition to the antibody-producing plasma cells that produce the autoantibodies, the disease process is also influenced by various other immune cells. In the different stages of LN, B, and T cells, as well as neutrophils and macrophages, play a key role in the progression and maintenance of renal injury [11–14]. While some immune cells are directly involved in pathogenesis, others are involved in disease progression, for example by secreting pro-inflammatory and profibrotic cytokines. In LN, these inflammatory factors lead initially to acute kidney injury (AKI), but ultimately to chronic kidney disease (CKD), which can progress to end-stage renal disease (ESRD) [15]. Currently, anti-inflammatory therapies such as glucocorticoids like prednisone; anti-malarials or immunosuppressants like cyclophoshamide, azathioprine, mycophenolate mofetil, and cyclosporine; or removal of harmful autoantibodies using plasmapheresis are used in the therapy of LN [16]. As targeted therapies, B-cell depletion with rituximab are still used [16] and targeting of other immune cells such as neutrophils, e.g., neutrophil extracellular traps, is discussed [17]. However, macrophage-specific therapies are not yet available. To target macrophages therapeutically, a better understanding of the role of these cells and individual macrophage subpopulations in disease is essential. Inflammatory cells, as well as renal cells, interact with macrophages via interleukins, chemokines, and growth factors, allowing them to differentiate into subtypes with different characteristics [18–21]. Macrophages can differentiate into a variety of subtypes and can be broadly divided into two groups: M1 (classically activated) pro-inflammatory macrophages and M2 (alternatively activated) immunomodulatory and more anti-inflammatory macrophages [22]. M2 macrophages can be further subdivided into M2a (anti-inflammatory, wound healing), M2b (immunoregulatory, pro-infective), M2c (immunosuppressive, phagocytic, and involved in tissue remodeling), and M2d (tumor progression, angiogenesis) [23]. The first classification of macrophages was proposed by Mantovani et al. [22]. The study of macrophage populations in tissue sections is challenging because suitable markers that can be detected with antibodies do not exist for all subpopulations. However, by combining different antibodies, at least some subpopulations of macrophages can be investigated. Since macrophage subpopulations have different roles in inflammatory response and repair processes they have an impact on the progression of LN [21]. It is difficult to reduce the finding to a single cell type with a single effect, as often the ratio between the phenotypes of, e.g., macrophages also plays a role in the final outcome of the effect [24]. In a previous study, we demonstrated that the number of specific macrophage populations in kidney biopsies from adult LN patients differs according to lupus class and that M2c-like macrophages are the predominant macrophages [25]. Data on early macrophage inflammation in LN are lacking and it is unclear whether class-specific macrophage populations are similarly distributed in pediatric cases. Therefore, in this study, 20 pediatric patients with LN were analyzed and compared to the results of the study with adult LN patients. In addition, to investigate whether there is an LN-specific inflammatory cell signature, T cells, B cells, and granulocytes in 4 additional pediatric inflammatory kidney diseases were analyzed by multiple immunofluorescence microscopy and compared with age-matched zero-time biopsies as controls. Finally, the inflammatory response was correlated with clinical and histopathologic renal changes.

## Methods

### Renal tissue specimens

In our study, we analyzed macrophage polarization and inflammatory cells in 73 pediatric cases with an age range of 1–17 years including 20 patients with lupus nephritis allocated to SLE classes II-IV according to ISN/RPS [2]. For pediatric controls with other kidney diseases, we included 11 cases with hemolytic uremic syndrome (HUS, including 8 cases with aHUS and 3 with STEC-HUS), 11 cases with membranoproliferative glomerulonephritis (MPGN), 15 cases with post-infectious glomerulonephritis (PI-GN, 9 streptolysin-positive and 7 streptolysin-negative), and 9 cases with ANCA-associated pauci-immune glomerulonephritis (PAUCI). Zero-biopsies from pediatric donor kidneys (*n* = 7) were used as healthy controls. All pediatric patients were treated at the Children’s Hospital of the FAU Erlangen, and the diagnoses were made by expert nephropathologists at the Department of Nephropathology (KA, MBH). An overview of the composition and clinical data of the collective of pediatric kidney patients is provided in Table 1. For comparison, we reevaluated data on macrophage polarization in adult patients with LN previously published by Olmes et al. [25].

**Table 1:** Characteristics of pediatric LN cohort

	Lupus ISN/RPS classes			
	Class II	Class III^a^	Class IV	Class V
Number of patients [*n*]	4	6	6	4
Age of Patients [years]	12.8 ± 0.8	14.2 ± 1.6	15.7 ± 1.3	13.5 ± 1.5
Male [*n*]	1	1	0	1
Female [*n*]	3	5	6	3
Hypertensive patients [%]	25	0	50	50
Diabetic patients [%]	0	0	0	0
Serum creatinine [mg/dl]	0.7 ± 0.2	0.5 ± 0.1	0.9 ± 0.3	1.9 ± 2.3
Serum urea [mg/dl]	34.8 ± 10.9	20.7 ± 6.3	51.0 ± 21.0	59.8 ± 62.0
GFR after Schwartz [ml]	104.6 ± 26.2	126.0 ± 27.3	84.5 ± 26.3	98.6 ± 56.6
Proteinuria [g/m^2^/24 h]	0.16 ± 0.06	0.79 ± 0.85	1.79 ± 1.93	2.93 ± 2.64
Hematuria [*n*/µl]	10.0 ± 10.0	485.0 ± 602.6	408.3 ± 471.0	920.0 ± 1433.6
Glomerular injury score [score 0–4]	1.6 ± 0.3	2.1 ± 0.2	2.4 ± 0.4	2.5 ± 0.9

^a^Including 2 cases diagnosed for both classes III and V but listed only in class III due to the presence of activity. If variables are not expressed as a single value, data were shown as mean ± SD

### Multiple immunofluorescence staining

For all co-localization studies, kidneys were fixed in formalin, embedded in paraffin, and cut into sections of 2 µm. Antigen retrieval was done using target retrieval solution pH 6 (Dako Deutschland GmbH, Hamburg, Germany) and cooking in a pressure cooker (Biocare Medical, Pacheco, CA, USA) for 2.5 min at 110 °C. After blocking with normal goat serum and 1% blotto sections were incubated overnight at 4 °C using the following antibodies diluted in 1% BSA in 50 mM Tris(hydroxymethyl) aminomethan pH 7.6: iNOS, a rabbit polyclonal antibody against human iNOS (Abcam plc, Cambridge, UK); CD68, a mouse monoclonal IgG3 antibody against human CD68 (Dako Deutschland GmbH, Hamburg, Germany); CD163, a mouse monoclonal IgG1 antibody against human CD163 (Novocastra, Leica Biosystems Newcastle Ltd; Newcastle, UK); CD206, a mouse monoclonal IgG1 antibody against human CD206 (Abnova, Jhongli City, Taiwan); CD3, a monoclonal rat antibody against human CD3 (Bio-Rad AbD Serotec GmbH, Puchheim, Germany); CD20, a monoclonal mouse IgG2a antibody against human CD20 and MPO, a polyclonal rabbit antibody against myeloperoxidase (Abcam plc, Cambridge, UK). Detailed information on used antibodies including immunoglobulin classes, dilutions, and purchase numbers are listed in Supplemental Table 1. Three different cocktails of primary antibodies were used: (1) anti-CD68, anti-CD206, anti-CD3, and anti-CD20; (2) anti-CD68, anti-CD163, and anti-iNOS; (3) anti-MPO. Negative controls for immunostaining included either deletion or substitution of the primary antibody with equivalent concentrations of an irrelevant murine monoclonal antibody or pre-immune rabbit IgG. After washing in 50 mM Tris(hydroxymethy)aminomethan pH 7.6 supplemented with 150 nM NaCl and 0.01% Tween 20, sections were incubated with the following secondary antibodies: respectively, a goat anti-mouse IgG1 antibody conjugated to Cy3 (Dianova GmbH, Hamburg, Germany); a goat anti-mouse IgG3 antibody conjugated to Alexa Fluor 488 (Dianova); a donkey anti-rat IgG antibody conjugated to Alexa Fluor 647 (Invitrogen, Carlsbad, CA, USA). Detailed information on used secondary antibodies including dilutions and purchase numbers are listed in Supplemental Table 2.

**Table 2:** Characteristics of pediatric controls with inflammatory kidney disease and zero-time biopsies

	**Controls**				
	HUS	MPGN	PI-GN	PAUCI	Zero-biopsies
Number of patients [*n*]	11	11	15	9	7
Age of patients [years]	5.8 ± 4.3	10.4 ± 5.1	8.4 ± 4.4	13.1 ± 2.0	14.7 ± 2.5
Male [*n*]	3	6	9	5	7
Female [*n*]	8	5	6	4	0
Hypertensive patients [%]	100	36.4	40.0	22.2	0
Diabetic patients [%]	0	0	0	0	0
Serum creatinine [mg/dl]	4.1 ± 4.4	1.3 ± 1.6	1.3 ± 1.6	4.4 ± 4.9	1.0 ± 0.6
Serum urea [mg/dl]	164.1 ± 150.4	62.6 ± 42.3	62.6 ± 42.3	81.2 ± 52.3	14.8 ± 6.3
GFR after Schwartz [ml]	25.6 ± 25.0	82.0 ± 39.5	71.3 ± 41.7	38.0 ± 27.9	84.3 ± 28.0
Proteinuria [g/m^2^/24 h]	2.0 ± 1.9	2.5 ± 1.9	2.2 ± 2.2	1.1 ± 1.5	n.d
Hematuria [*n*/µl]	278 ± 487	1178 ± 2935	7683 ± 11,560	2521 ± 3869	n.d
Glomerulosclerosis index [score 0–4]	2.8 ± 0.6	2.3 ± 0.5	2.9 ± 0.5	3.3 ± 0.5	0.6 ± 0.6

If variables are not expressed as a single value, data were shown as mean ± SD. *n.d.* no data available

Finally, sections were digitalized with a slide scanner (Zeiss Z1, Zeiss, Oberkochen, Germany) and analyzed using Qupath software (version 0.2.3) [26].

### Quantitative and qualitative evaluation of macrophages, MPO + , CD3 + , and CD20 + cells in renal biopsies

CD68 + /CD206 − , CD68 + /CD206 + , CD68 + /CD163 − , and CD68 + /CD163 + cells and total CD68 + cells as well as CD3 + , CD20 + , and MPO + cells were counted manually in the whole slide scans of renal biopsies and Qupath software at × 400 magnification. After evaluation of section area, inflammatory cell counts were normalized by calculation of positive cells per mm^2^.

To investigate the effects of macrophage polarization on glomerular inflammation and crescent formation, we focused on biopsies from patients with ISN/RPN class IV juvenile LN, as this group was the largest in the juvenile LN cohort and crescents were present. In this group, a total of 75 individual glomeruli of 6 biopsies were evaluated by counting the different macrophage subpopulations and other inflammatory cells (CD3 + , MPO + , and CD20 +) per glomerular section in the same glomerulus of the same or subsequent section. In crescentic glomeruli, inflammatory cells were also differentiated between those located in the tuft and those located in the crescent.

### Histopathological evaluation

Changes in glomerular morphology including endothelial injury, mesangial matrix accumulation, sclerosis, and crescent formation were evaluated by a semi-quantitative glomerular injury score (GIS) using PAS-stained paraffin sections. Glomeruli without changes were graded with 0, glomeruli with changes present in up to 25% of the tuft = score 1, glomeruli with changes present in 26–49% of the tuft = score 2, glomeruli with changes present in in 50–75% of the tuft = score 3, glomeruli with changes present in more than 75% of the tuft area = score 4.

### Evaluation of retrospective clinical data

Clinical parameters from patients were retrospectively acquired from the time point of biopsy collection. The following parameters were included for correlation analysis of the evaluated macrophage subpopulations, inflammatory cells, and injury scores using SPSS software: patient age, hypertension, diabetes, proteinuria, hematuria, eGFR [27], serum creatinine, and serum urea.

### Statistical analyses

After testing for normal distribution of values using the Kolmogorov–Smirnov test and finding that many data sets are not normally distributed, data were analyzed using the Kruskal–Wallis test and Dunn’s multiple comparison test as a post hoc test for comparison of SLE ISN-RPS classes and comparison with other pediatric inflammatory kidney diseases. In all tests, *p* < 0.05 were considered statistically significant. Data are presented as bars representing the mean ± SEM. Spearman’s test was used to test the correlation of renal macrophage, T-cell, B-cell, and neutrophil infiltration with kidney injury scores and clinical data. Statistical analyses were performed using SPSS for Windows software (version 28.0 SPSS, IBM, Munich, Germany) or GraphPad Prism for Windows software (version 5.02, GraphPad software Inc., San Diego, CA, USA). Hierarchical clustering analysis (Euclidean distance, complete-linkage) using data for inflammatory cell counts per cortex area was performed in the R software environment v. 4.3.1 (R Core Team (2023). R: A language and environment for statistical computing. R Foundation for Statistical Computing, Vienna, Austria. URL http://www.R-project.org/) relying on the pheatmap package v. 1.0.12 (Kolde R (2019). _pheatmap: Pretty Heatmaps, https://CRAN.R-project.org/package=pheatmap). Prior to cluster analysis, 31 patients with missing values in macrophage or inflammatory cell counts have been excluded and values of inflammatory cell counts per area have been mean-centered.

## Results

### Characteristics of pediatric SLE patients with ISN/RPS classes II–V and controls

This study included 20 pediatric SLE patients with LN ISN/RPS classes II–V. Biopsies from patients with HUS, MPGN, PI-GN, PAUCI, and zero-time transplants served as control groups. Most of the pediatric LN patients were female (*n* = 17) and only 3 male and hereby differed to sex distribution in the control groups that either had a balanced sex ratio or even contained more males (Tables 1 and 2). The mean age in pediatric SLE patients ranged from 12.8 to 15.7 years in the different ISN/RPS classes (Table 1), whereas the pediatric cases in the HUS and PI-GN control groups were significantly younger (Table 2). The percentages of reported hypertension ranged in the ISN/RPS lupus classes from 0 (class III) to 50% (class IV) (Table 1). In controls, none of the zero-biopsies had a hypertensive donor, while in the inflammatory kidney disease controls the percentage of hypertensive patients ranged from 22.2% in PAUCI to 100% in the HUS group (Table 2). None of the patients was diagnosed with diabetes in any of the groups studied.

Regarding the clinical data on kidney function, serum creatinine levels in pediatric LN were only moderately elevated with highest mean in the ISN/RPS class V (1.9 ± 2.3 mg/dl). In contrast, creatinine levels in control groups with inflammatory kidney diseases reached higher mean values with the highest in patients diagnosed with PAUCI (4.4 mg/dl, Table 2). However, the variability in serum creatinine levels was high in all groups.

Similarly, serum urea was also moderately elevated and glomerular filtration rate was hardly reduced in pediatric LN and we observed more severe impairment of kidney function in controls with inflammatory kidney diseases (Tables 1 and 2). Proteinuria and hematuria showed again high variability and were relatively low compared to other inflammatory kidney disease controls (Tables 1 and 2). Glomerular injury score varied within groups but mean values were lowest in zero-time controls and pediatric LN class II, while in all other groups, the mean GIS was above 2 (Tables 1 and 2). The renal changes assessed by GIS were comparable in the presented juvenile LN cohort compared to the former adult LN study (mean age 34–40 years, Supplemental Table 3).

### Comparison of macrophage subpopulations in ISN/RPS classes in pediatric versus adult patients

**Fig. 1:**
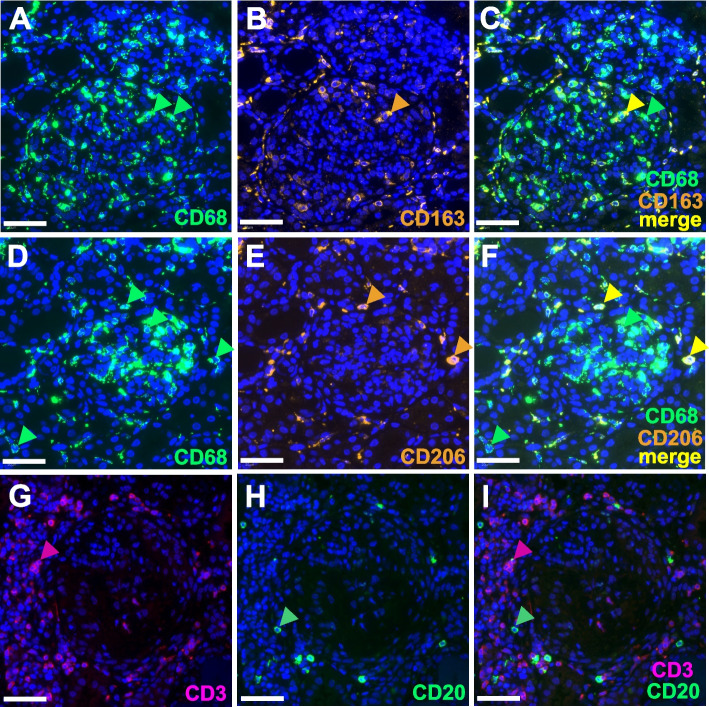
Immunofluorescence staining of inflammatory cells in pediatric kidneys. **A** CD68 + macrophages. **B** CD163 + cells. **C** Merge of CD68 + and CD163 + cells representing M2c-like macrophages. **D** CD68 + macrophages. **E** CD 206 + cells. **F** Merge CD68 + and CD206 + cells, representing M2a-like macrophages. **G** Renal T lymphocytes (CD3 + cells). **H** Renal B lymphocytes (CD20 + cells). **I** B and T Lymphocyte overlap (CD3 + and CD20 + cells. Scale bar represents 50 µm

**Fig. 2:**
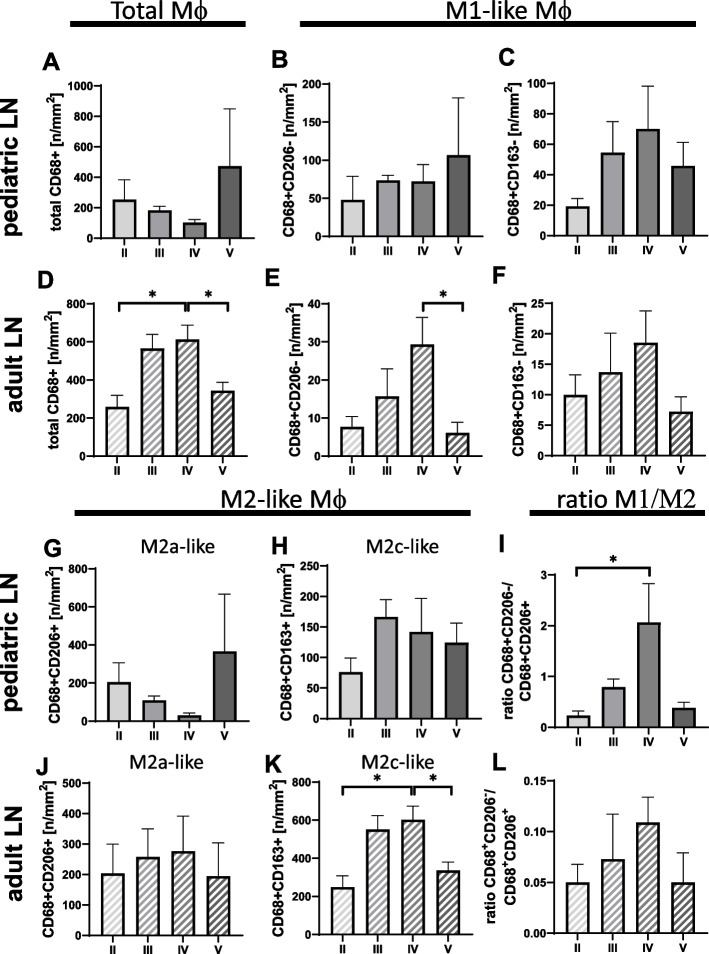
Distribution of macrophage subsets in different ISN/RPS classes. **A** CD68 + macrophages in pediatric LN patient groups representing the total macrophages. **B** CD68 + CD206 − cells (M1-like macrophages) in pediatric LN patients. **C** CD68 + CD163 − cells (M1-like macrophages). **D** CD68 + as total macrophages in adult LN patients with a significant difference between groups II, V, and IV. **E** CD68 + CD206 − cells (M1-like macrophages) with a significant difference between groups IV and V. **F** CD 68 + CD163 − cells ((M1-like macrophages) (**E** and **F** in adult LN patients). **G**–**I** Showing results for pediatric LN patients, **G** CD68 + CD206 + cells (M2a-like macrophages), **H** CD68 + CD163 + cells (M2c-like macrophages), **I** Ration of CD68 + CD206 − vs CD68 + CD206 + cells (M1-like macrophages and M2a-like macrophages) with a significant difference between groups II and IV. **J**–**L** representing adult LN results. **J** CD68 + CD206 + cells (M2a-like macrophages), **K** CD68 + CD163 + cells (M2c-like macrophages), with a significant difference between groups II, V, and IV. **L** Ration of CD68 + CD206 − vs CD68 + CD206 + cells (M1-like macrophages and M2a-like macrophages). (*p < 0.05; **p < 0.01)

Immunofluorescence staining was used to examine both the total number of CD68-positive macrophages and different subpopulations by combining with markers for M2a (CD206) and M2c (CD163) (Fig. 1A–F). Unfortunately, detection of iNOS as a marker of M1-like macrophages in immunofluorescence was not successful, so we alternatively determined CD68 + CD163 − and CD68 + CD206 − cells as M1-like macrophages (Fig. 1C, F, green arrowheads). In addition, CD3-positive T cells, CD20-positive B cells (Fig. 1G–I), and MPO-positive neutrophils (data not shown) were assessed in the kidney biopsies of pediatric patients with lupus nephritis. Since we wanted to investigate whether macrophage infiltration in kidney biopsies with LN differs in pediatric compared to adult patients, we divided the cases of pediatric LN according to ISN/RPS classes and compared them with an old data set in which we had investigated macrophage infiltration in mainly adult LN patients (Olmes et al.) [25]. For this purpose, we removed 3 pediatric LN cases to receive an exclusively adult LN patient collective. First, we analyzed the total CD68 + macrophage infiltration in all 4 SLE-ISN/RPS classes studied in both pediatric and adult LN. Interestingly, the mean numbers of total macrophages was in pediatric LN patients ranged between 102 and 472 cells per mm^2^ in the different LN ISN/RPS classes but showed high variation especially in classes II and V (Fig. 2A). In adult patients with class II and IV LNs, the total number of macrophages was approximately three times higher than in juveniles (Fig. 2A, D). Due to the low number of cases, especially in pediatric LN patients, significant differences between LN ISN/RPS classes could only be described in the cohort of adult LN patients, and here the infiltration with total macrophages was significantly higher in LN class IV compared to classes II and V (Fig. 2D). For M1-like macrophages, we examined two different populations, the CD68 + CD206 and the CD68 + CD163 cells. While the number of CD68 + CD206 macrophages in pediatric LN patients showed only minor differences when comparing the different ISN/RPS classes, this cell population was more than 3 times more abundant in class IV than in classes II and V in adult LN (Fig. 2B, E). In contrast, CD68 + CD163 − macrophages showed a similar distribution pattern across LN classes in pediatric and adult patients with the highest number in class IV, but were approximately twice as abundant in pediatric as in adult LN (Fig. 2C, E).

Although the counts of M2a-like macrophages did not show significant differences when comparing LN classes in either juveniles or adults. However, the distribution was quite different. While most M2a-like macrophages were detected in classes II and V in pediatric LN (Fig. 2G), this subpopulation was similarly abundant in all classes in adults (Fig. 2J). In contrast, the distribution of M2c-like macrophages with high numbers in classes II and IV was very similar in pediatric and adult patients, but this subpopulation was almost 4 times more abundant in kidneys from adult LN patients compared to children (Fig. 2H, K). In contrast, the ratio values of M1-like/M2a-like macrophages in pediatric LN patients were significantly higher in class IV compared to class II and up to 10 times higher compared to adult LN patients (Fig. 2I, L).

### M1-like macrophages dominate in glomeruli of pediatric LN ISN/RPN class IV patients and are associated with MPO + neutrophilic granulocyte and CD3 + T-cell numbers

**Fig. 3:**
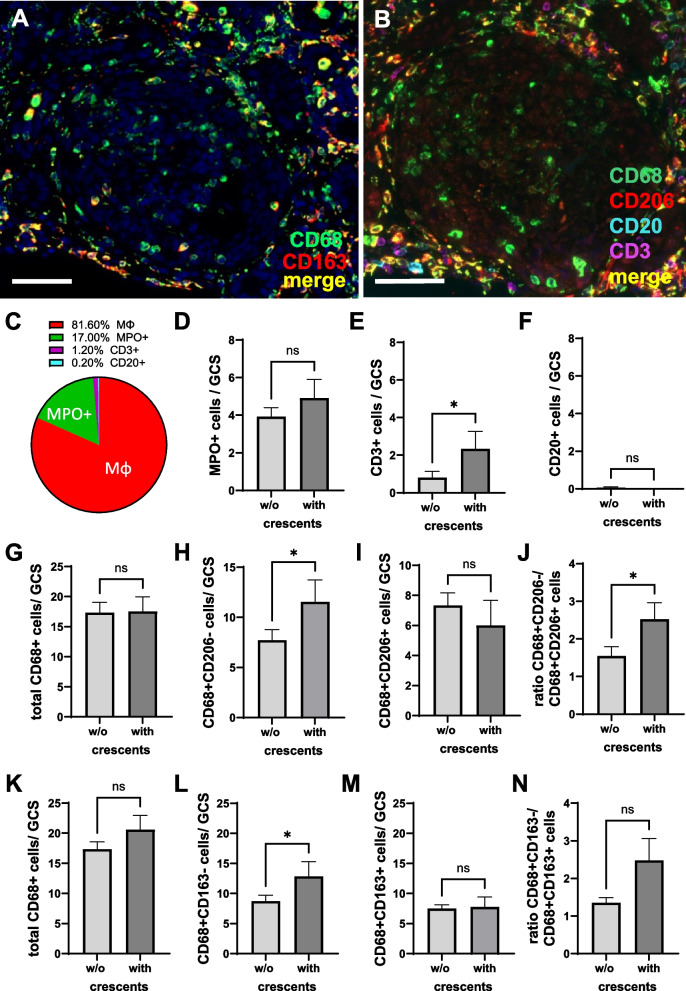
Distribution of macrophage-subtypes in pediatric LN ISN/RPN class IV in glomeruli with and without (w/o) crescents. **A** Representative multiple immunofluorescence staining for CD68 + and CD163 + cells using biopsies from patients with ISN/RPN class IV. **B** Multiple immunofluorescence staining for CD68 + , CD206 + CD20 + , and CD3 + cells using the same glomerulus imaged in (**A**) in another section. **C** Pie chart showing the distribution of different inflammatory cells in glomeruli from pediatric LN biopsies with ISN/RPN class IV. **D** MPO + cells /glomerular cross-section (GCS). **E** CD3 + T-cells / GCS. **F** Glomerular CD20 + B-cells. **G** Glomerular total CD68 + macrophages per GCS (first antibody panel). **H** Glomerular CD68 + CD206 − M1-like macrophages/ GCS. **I** Glomerular CD68 + CD206 + M2a-like macrophages. **J** Ratio of glomerular CD68 + CD206 − /CD68 + CD206 + macrophages. **K** glomerular total CD68 + macrophages per GCS (second antibody panel). **H** Glomerular CD68 + CD163 − M1-like macrophages/ GCS. **I** Glomerular CD68 + CD163 + M2c-like macrophages. **J** Ratio of glomerular CD68 + CD163 − /CD68 + CD163 + macrophages. (*p < 0,05). Scale bar represents 50 µm

Next, we investigated whether macrophage subpopulations influence glomerular inflammation and crescent formation. We focused on lupus nephritis ISN/RPS class IV because this is the LN group with the most cases and includes glomeruli with crescent formation. Representative images of glomeruli from this group show that M1-like macrophages (CD68 + CD206 − , Fig. 3A; CD68 + CD163 − , Fig. 3B, green staining) are the dominant macrophage subpopulation. Macrophages were the most abundant inflammatory cells in the glomeruli with a mean of 81.6%, followed by MPO + neutrophilic granulocytes, which represented 17% of the glomerular inflammatory cells (Fig. 3C, D). CD3 + cells were rarely found in the glomeruli examined, both in the glomerular tuft and in the crescents (Fig. 3B, C). However, CD3 + T cells were more abundant in glomeruli with crescents (Fig. 3E), whereas the number of MPO + cells was comparable in glomeruli with and without crescents (Fig. 3D). CD20 + B cells were almost completely absent in the glomeruli (Fig. 3B, F). M1-like macrophages were present in the glomeruli of juvenile LN class IV patients, as evidenced by higher numbers of CD68 + CD206 − versus CD68 + CD206 + macrophages (Fig. 3G–J) and CD68 + CD163 − versus CD68 + CD163 + macrophages (Fig. 3K–N). Interestingly, this subpopulation was more abundant in crescentic glomeruli (Fig. 3H, L, J). Spearman correlation analysis showed that the number of glomerular MPO + neutrophilic granulocytes was significantly correlated with the number of M1-like macrophages, i.e., mainly CD68 + CD206 − (*r* = 0.608) and CD68 + CD163 − (*r* = 0.456), but not with the number of M2-like macrophages. Accordingly, MPO + cells also correlated with the ratio of glomerular CD68 + CD206 − /CD68 + CD206 + (*r* = 0.451) and the ratio of CD68 + CD163 − /CD68 + CD163 + macrophages (*r* = 0.318) (Suppl. Figure 1A). However, the ratio of M1-like to M2-like macrophage subpopulations in the glomerular tuft did not differ from that in the crescent (Suppl. Figure 1B).

### Compared to other inflammatory kidney diseases, patients with lupus nephritis have low macrophage, T-cell, B-cell and neutrophil infiltration in pediatric kidney biopsies

**Fig. 4:**
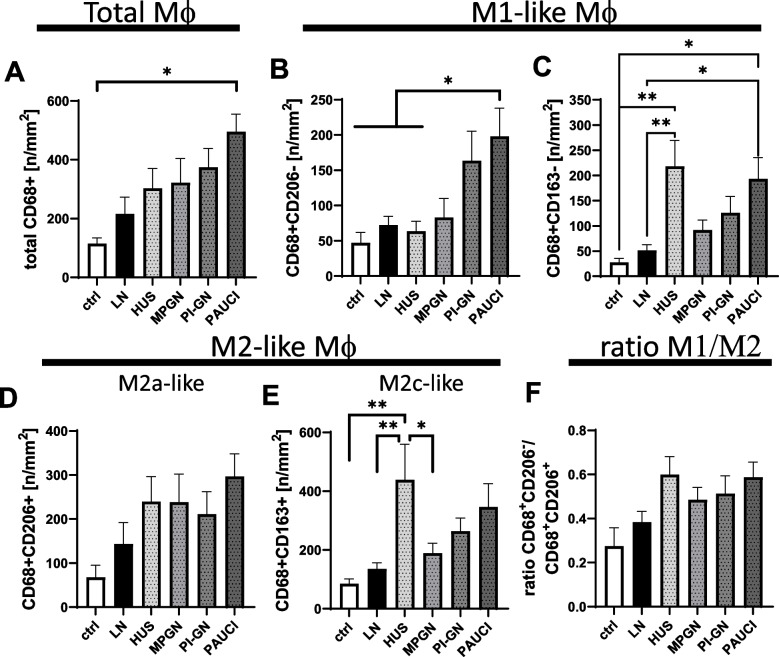
Distribution of macrophage-subtypes in pediatric LN and a pediatric control group consisting of HUS, MPGN, PI-GN and PAUCI patients. **A** CD68 + total macrophages in the inflammatory kidney in pediatric patients with significantly higher in PAUCI patients compared to the LN and control group. **B** CD68 + CD206 − M1-like macrophages significantly higher in pediatric PAUCI patients compared to all other study groups. **C** CD68 + CD163 − M1-like macrophages significantly higher in PAUCI than in LN patients. **D** CD68 + CD206 + M2a-like macrophages, significantly higher in PAUCI than in LN patients. **E** CD68 + CD163 + M2c-like macrophages, significantly higher in HUS pediatric patients compared to MPGN, LN patients and our pediatric control group. **F** Ratio of CD68 + CD206 − and CD68 + CD206 + (M1-like and M2a-like macrophages) in pediatric patients. (*p < 0.05; **p < 0.01)

In addition to a control group of healthy pediatric patients, we also studied renal biopsies from pediatric patients with other inflammatory kidney diseases such as hemolytic uremic syndrome (HUS), membranoproliferative glomerulonephritis (MPGN), postinfectious glomerulonephritis (PI-GN), and ANCA-associated pauci-immune glomerulonephritis (PAUCI). Compared with renal biopsies from patients with other inflammatory kidney diseases, the LN group has the lowest number of total macrophages in the renal cortex (Fig. 4A). However, due to the relatively large variance and small number of cases, only the comparison with the PAUCI group, which had almost 5 times as many macrophages on average, reaches the significance level (Fig. 4A). When comparing individual macrophage subpopulations, the number of macrophages in the LN group (containing all LN classes examined) was only slightly and never significantly increased compared to the control group (Fig. 4). Both CD68 + CD206 − and CD68 + CD163 − M1-like macrophages were significantly increased in the PAUCI group compared to the LN group (Fig. 4B, C). In addition to the PAUCI group, CD68 + CD206 − macrophages were also high in the PI-GN group and CD68 + CD163 − macrophages were significantly higher in the HUS group compared to healthy control and LN (Fig. 4B, C). Despite up to five times higher mean numbers of M2a-like macrophages compared to healthy controls, no significant differences can be described between investigated groups of inflammatory kidney disease (Fig. 4D). In contrast, M2c-like macrophages were significantly and approximately threefold more abundant in kidney biopsies from HUS patients compared to LN, MPGN, and controls (Fig. 4E). Compared to the control group, the mean M1/M2 ratio in LN was only slightly higher, and the mean ratios in the other inflammatory kidney diseases were on average about twice as high as in the control group, but did not reach significance (Fig. 4F).

**Fig. 5:**
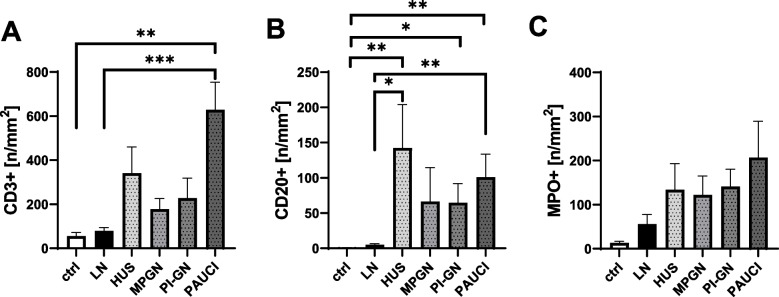
T-, B-lymphocytes, and neutrophilic granulocytes in pediatric study groups. **A** CD3 + T-lymphocytes in pediatric patients. Significantly higher in PAUCI patients compared to the LN and control group. **B** CD20 + B-lymphocytes significantly higher in PAUCI and HUS compared to LN patients as well as post-infectious patients compared to our control group. **C** MPO + cells resembling neutrophils. (*p < 0.05;**p < 0.01)

The relatively low macrophage infiltration in pediatric LN is also accompanied by low infiltration with CD3-positive T cells, CD20-positive B cells, and MPO-positive neutrophilic granulocytes compared to controls (Fig. 5). A particularly high infiltration with CD3-positive T cells and CD20-positive B cells was detected in the PAUCI group, each of which was significantly increased compared to the control and LN groups (Fig. 5A, B). T and B cells were also significantly more abundant in the kidney of pediatric HUS patients compared with the control and LN groups, but only B cells reached the significance level (Fig. 5A, B). Neutrophils were more than tenfold higher on average in MPGN, PI-GN, and PAUCI, but never reached the significance level when comparing the groups (Fig. 5C).

### Different pediatric inflammatory kidney diseases do not cluster according to their inflammatory marker profiles

**Fig. 6:**
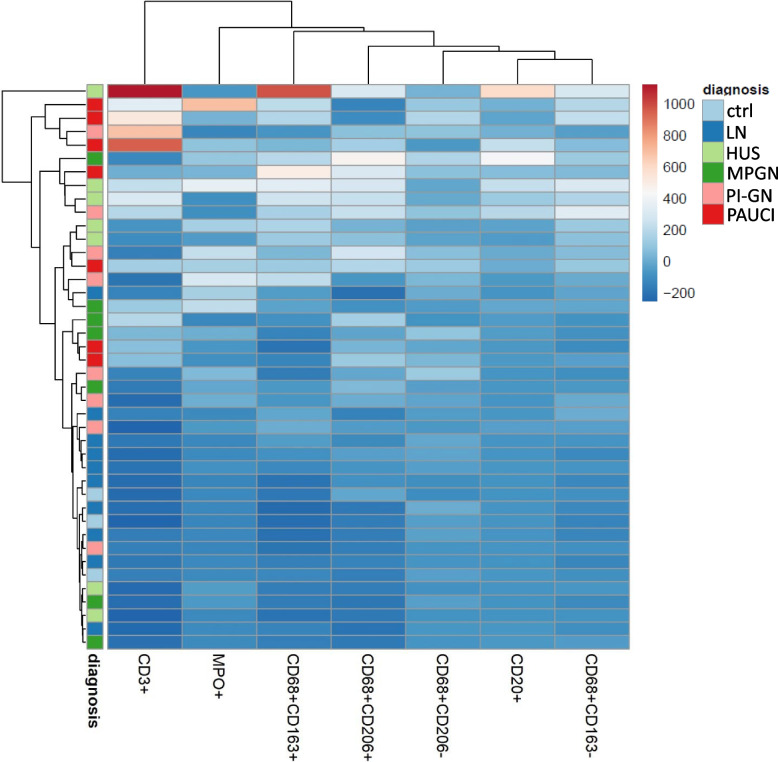
Clustering analysis of the abundance of inflammatory cells in different inflammatory kidney diseases in pediatric patients. Heatmap of the numbers of CD68 + CD163 + , CD68 + CD206 + , CD68 + CD163 − , CD68 + CD206 − , CD3 + , CD20 + , and MPO + cells in the analyzed patients (red: high number; blue: low number). The color-coded bar corresponding to the different rows (patients) indicates the diagnosis

We used clustering analysis to explore whether the studied juvenile renal diseases grouped on the basis of the distribution of macrophage subpopulations and other inflammatory cells. However, with respect to the inflammatory cell infiltrate, some cases per disease clustered in a specific range, while others with the same diagnosis did not (Fig. 6). For example, as expected from the individual analyses, many cases with PAUCI and HUS clustered together showing elevated inflammatory cell counts, while many LN and controls showed low inflammation and clustered together. Overall, however, the number of different inflammatory cells showed high variability and samples with the same disease did not cluster together (Fig. 6).

### The extent of inflammatory cell infiltration often correlates with renal function and damage

**Fig. 7:**
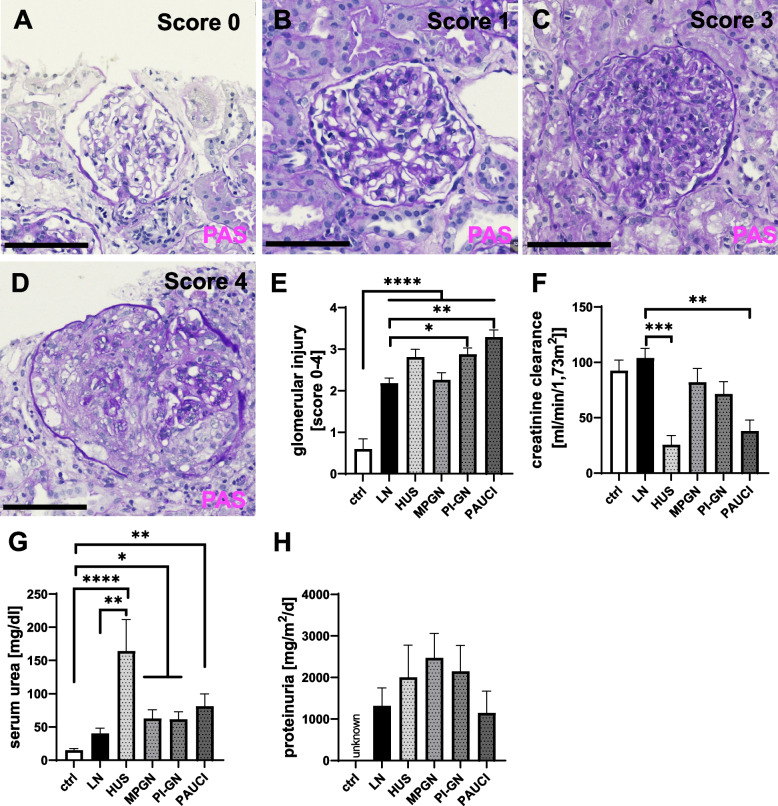
Glomerular injury score (GIS), serum creatinine, serum urea, and proteinuria used to evaluate the extent of kidney injury in pediatric patients with inflammatory kidney diseases. **A** GIS score 0. **B** GIS score 1. **C** GIS score 3. **D** GIS score 4. **E** GIS in pediatric patients. Significantly higher in all groups compared to the control group, significantly higher in PI-GN and PAUCI patients than in LN patients. **F** Serum creatinine is significantly higher in LN Patients compared to HUS and PAUCI patients. **G** Serum urea is significantly higher in pediatric HUS patients compared to LN patients and the control group. The control group as significantly lower levels than all study pathological groups. **H** Proteinuria in our study groups

The extent of morphologic renal damage in the different pediatric inflammatory kidney diseases was determined using a glomerulosclerosis score ranging from 0 to 4 (Fig. 7A–D) and showed significant glomerulosclerosis in all groups compared with controls (Fig. 7E). Renal function, as measured by eGFR after Schwartz and serum urea, was barely impaired in LN compared to controls (Fig. 7F, G). The most severe impairment of renal function was observed in the HUS group (Fig. 7F, G). In contrast, proteinuria was present in all groups studied, with a large variance within groups and no significant difference between groups (Fig. 7H).

**Fig. 8:**
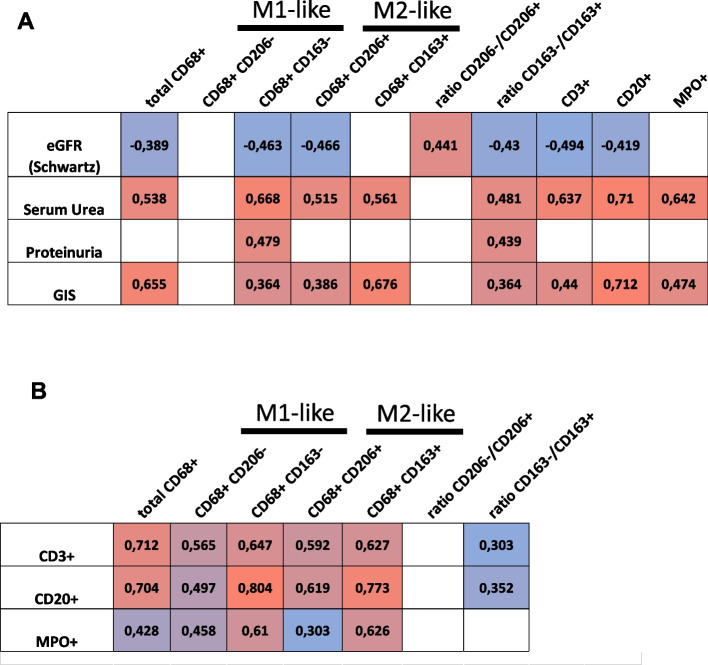
Correlation of clinical parameters with inflammatory cell infiltrates in pediatric inflammatory kidney disease. **A** Significant (*p* < 0.05) correlations between the eGFR, serum urea, and proteinuria as markers of kidney function with the cell counts of inflammatory cells including macrophage subtypes. **B** Significant (*p* < 0.05) correlations between cortical CD3 + T-cells, CD20 + , and MPO + cells with the cell counts of macrophage subtypes and subtype ratios detected in all pediatric inflammatory kidney disease groups, as assessed by Spearman’s test

Finally, we correlated the cortical infiltration of different inflammatory cell populations with renal functional parameters and glomerular injury, independent of the particular pediatric kidney disease (Fig. 8). Almost all inflammatory cells showed a significant positive correlation with renal function, measured as eGFR and serum urea. CD68 + CD163 − as well as CD3 + , CD20 + , and MPO + cells correlated particularly strongly with serum urea concentrations (Fig. 8A). There were less obvious correlations with proteinuria, but these also showed an association with M1-like macrophages (CD68 + CD163 −). Glomerular injury score correlated positively with total macrophages, but especially with M2c-like macrophages and CD20 + B cells (Fig. 8A). The frequency of CD3 + T cells, CD20 + B cells, and MPO + neutrophilic granulocytes in the renal cortex correlated significantly, but to different degrees, with the different macrophage populations (Fig. 8B). CD3 + correlated most strongly with the total number of macrophages (*r* = 0.712), CD68 + CD163 − (*r* = 0.647), and CD68 + CD163 + (*r* = 0.627) (Fig. 8B). A similar association was observed for CD20 + B-cells, which, however, correlated most strongly with CD68 + CD163 − macrophages (Fig. 8B). In contrast, the number of MPO + cells correlated primarily with the number of CD68 + CD163 − (*r* = 0.610) and CD68 + CD163 + (*r* = 0.626) macrophages. Thus, CD68 + CD163 − macrophages play a special role in the inflammatory process as indicated by their strong association with other inflammatory cell types.

## Discussion

### Macrophage subpopulations in pediatric and adult patients with LN

It is well known that macrophages play an important role in the pathogenesis of LN [28–30], but less is known about macrophage subtypes in the pathogenesis of LN, especially in human LN. In a previous study, we showed that surprisingly, most macrophages in the kidneys of adult lupus patients expressed CD163 and were classified as predominantly M2-like macrophages [25]. We also demonstrated in adult LN that the number of cells in a given macrophage subpopulation differs as a function of ISN-RPN lupus class [25]. In this study, we investigated whether the polarization of macrophages in pediatric patients differs from that in adults. Here, we observed that the number of renal macrophages in kidneys from patients with pediatric LN was lower by about half compared to biopsies from adult LN patients. Interestingly, the distribution of abundance of total, CD68 + CD206 − , and CD68 + CD206 + macrophage subpopulations in kidneys from pediatric and adult LN patients were different in ISN-RPN lupus classes. Although the proportion of M2-like macrophages expressing CD163 and/or CD206 is also significantly higher than the proportion of M1-like macrophages in kidneys from most pediatric LN patients (ISN/RPN classes II, III, and V), M1-like macrophages were significantly higher in pediatric LN than in kidneys from adult patients with LN and even the dominant subpopulation in ISN/RPN class IV. In animal models of LN, pro-inflammatory M1-like macrophages have been shown to be particularly important at the onset of LN disease. MRL^lpr^ lupus mice transiently exposed to ischemia/reperfusion developed defective renal repair with expansion of M1-like macrophages leading to an earlier onset of LN [31]. This is consistent with the fact that in the early, active phase of lupus disease in MRL^lpr^ mice pro-inflammatory cytokines such as TNFα and IL-1 were increased, which are also involved in macrophage differentiation and can induce differentiation into M1-like macrophages [32]. In our pediatric LN cohort only in biopsies from patients with ISN/RPN class IV, M1-like macrophages were the dominant macrophage subtype in the cortex and the glomeruli. Although SLE may have been present for a longer period of time in our pediatric patients, these cases probably represent the onset of the disease rather than cases from adult LN patients and may explain the higher proportion of M1-like macrophages in pediatric compared to adult LN biopsies. This assumption is supported by the fact that only 2 biopsies with pediatric LN showed signs of chronic changes such as increased interstitial fibrosis and only one biopsy contained a fibrous crescent. The role of macrophage subtypes in the development of LN disease is still unclear. There are studies suggesting that either M1 or M2 macrophages play a more important role in the pathogenesis of LN. Stimulation with a TLR2 agonist allowed monocytes to differentiate primarily into M2-like macrophages and delayed disease development in the NZB/W lupus mouse model [33]. In contrast, transferring in vitro polarized M1-like macrophages in a murine lupus model aggravated glomerular injury [34]. In our study, CD68 + CD206 − and CD68 + CD163 − cells were more abundant in glomeruli with crescent formation. The higher numbers of CD3 + T-cells in crescentic glomeruli might be promoted by the pro-inflammatory effects of these M1-like macrophages. Another study showed that in NZB/W lupus mice treated with CTLA4Ig and anti-CD153, M2b macrophages were a marker for successful remission [13]. However, there is also evidence that CD163-positive macrophages are involved not only in healing after acute injury, but also in the progression of kidney disease. Glomerular crescents, typical of LN classes III and IV, contain mainly M2-like macrophages, and the number of CD163 − and CD206-positive macrophages correlated positively with the activity index [35]. CD163-positive macrophages are thought to be anti-inflammatory, but studies in an LN mouse model have shown that these macrophages exhibit Bach1-mediated reduced expression of heme oxygenase-1, which can also be induced in vitro by stimulation with type 1 interferons [36]. Kishimoto et al. therefore suggested that these CD163-positive macrophages are dysregulated and tend to be pro-inflammatory [36]. The human study [35] on the role of macrophage subpopulations in crescent formation is performed in adult patients. Our data in pediatric LN patients show that crescent formation in the pediatric setting is associated with the presence of M1-like macrophages, and this may represent an early phase of the disease or be specific to the age of the patient group. Proinflammatory M1-like macrophages may also attract CD3 + T cells, which were found in higher numbers in crescentic glomeruli than in noncrescentic glomeruli in our study. In all glomeruli, both M1-like macrophage subpopulations correlated significantly with MPO + neutrophilic granulocytes due to their destructive nature. However, our data can only describe associations between these inflammatory cells, but cannot clarify which inflammatory cells are the first to enter the glomerulus and attract the other immune cells.

### Inflammatory response in pediatric LN compared to other pediatric inflammatory kidney disease

Surprisingly, the inflammation in our pediatric LN cases was quite low compared to the other pediatric inflammatory kidney diseases studied. Macrophages, neutrophils, T cells, and B cells were more abundant on average in all other diseases studied, although the comparisons did not reach the significance level due to the small number of cases. Therefore, we have not been able to describe a pattern of inflammatory cell infiltrate characteristic of each disease. M2-like macrophages, such as M2c-like and M2a-like macrophages, appear to be more abundant than M1-like macrophages in other kidney diseases. This has also been shown in several studies mainly in adult patients [37, 38]. Inflammatory cells contribute significantly to the development of pathological changes in renal diseases. Depending on the disease, the importance of each immune cell type certainly varies in pathogenesis. It is striking that the biopsies with PAUCI show the highest numbers for almost all immune cells. However, in our study with only small numbers of pediatric patients with inflammatory kidney disease, we could not confirm disease-specific inflammatory signatures. Furthermore, reports describing renal inflammation in pediatric patients are lacking.

### Relevance of inflammatory cell infiltration for renal function and damage

Macrophages appear to play a major role in the pathogenesis and progression of LN. The number of CD68-positive macrophages correlated significantly with serum creatinine and proteinuria in a study of LN patients [28] and interstitial CD68-positive macrophages were the best predictor for progression of LN [39]. In our previous study of adult patients with LN, we demonstrated the clearest correlation for renal function, as assessed by serum urea and creatinine, with M2-like macrophages, specifically CD163-positive M2c macrophages [25]. Since CD163-expressing macrophages are a potential source of soluble CD163 (sCD163), it is no surprise that sCD163 levels also correlated with LN disease activity, fibrinoid necrosis, and cellular crescents [40]. Another study suggested sCD163 as a marker for LN disease severity and an important indicator of poor prognosis in LN patients [41]. This fits well with our data that CD163-positive macrophages, in particular, correlate positively with glomerular injury and impaired kidney function in our study of pediatric inflammatory kidney diseases. This suggests an involvement of macrophage subpopulations in pathogenesis and progression of nephropathies. While M1-like macrophages in particular are considered to be pro-inflammatory and detrimental to functional kidney cells, this may not be limited to M1-like cells as CD163-positive cells may also exhibit pro-inflammatory properties [34]. M2-like macrophages are known to reduce inflammation. M2a macrophages, characterized by CD206, clear immune complexes, cellular debris, secrete anti-inflammatory cytokines such as IL-10, but also promote fibrosis by secreting TGF-ß [42]. CD163-positive M2c-like macrophages are capable of phagocytosis and play an important role in immune regulation and tissue remodeling [22]. Macrophages are also key effectors in secreting cytokines driving autoimmunity [43]. In addition to macrophages, many other immune cells are involved in the pathogenesis of inflammatory kidney disease. In our study, we showed that the number of T cells, B cells and neutrophilic granulocytes correlated with serum urea, as a marker of renal function and glomerulosclerosis. We observed the highest correlation with CD20-positive B cells. Autoantibodies are directly involved in the pathogenesis of several inflammatory kidney diseases such as LN and ANCA-associated pauci-immune GN (PAUCI) and can locally activate complement and hereby attract macrophages and neutrophils via C5a to the site of inflammation [44]. Surprisingly, we also found high numbers of CD20-positive cells in kidney biopsies of pediatric HUS patients. However, the pediatric HUS cases in our cohort were characterized by particularly severe renal damage and may not be representative. In our pediatric inflammatory kidney disease cohort, we also detected many neutrophils in the biopsies, which may be involved in the pathogenesis of kidney disease by direct cellular damage or by neutrophil extracellular traps [45]. Thus, the observed renal damage cannot be explained by macrophage infiltration alone, but probably results from the interaction of the different inflammatory cells.

### Limitations of the study

Cases of pediatric inflammatory kidney disease with renal biopsies are rare and therefore the numbers are infrequent. This is especially true for the individual ISN/RPS lupus classes. The classification of macrophages into M1- and M2-like macrophages is very simplified, as it is known that far more separate macrophage populations can be differentiated using analysis of central transcriptional regulators related to overall macrophage activation and regulators related to stimulus-specific programs [46]. The characterization of macrophage subtypes using surface markers on tissue sections is difficult due to the lack of suitable antibodies, so that only M1-like and M2-like macrophage populations could be described. To compare macrophage subpopulations in kidney biopsies from pediatric and adult patients with LN, data from a previous study using the same antibodies but a different detection method (IHC) were used.

## Conclusion

Although M1-like macrophages play a greater role in pediatric LN patients than in adult LN patients, M2-like macrophages appear to be key players and are also more abundant in other pediatric inflammatory kidney diseases. Interestingly, inflammation was relatively low in LN compared to other inflammatory kidney diseases in children. Not only macrophage subpopulations, but also other immune cells such as CD20-positive B cells correlated positively with the severity of kidney disease.

### Supplementary Information

**Additional file 1: Supplemental Table 1.** Characteristics of adult LN cohort. **Supplemental Table 2.** Primary antibodies used for immunofluorescence microscopy. **Supplemental Table 3.** Secondary antibodies used for immunofluorescence microscopy (IF).

**Additional file 2: Supplemental Figure 1.** Association of glomerular inflammatory cells with macrophage subtypes.

## Data Availability

Condensed anonymized data are available from the corresponding author on reasonable request.

## Abbreviations

eGFR: Estimated glomerular filtration rate
GN: Glomerulonephritis
HUS: Hemotytic uremic syndrome
MPO: Myeloperoxidase
MPGN: Membranoproliferative glomerulonephritis
PAUCI: Pauci immune glomerulonephritis
PI-GN: Post-infectious glomerulonephritis
ISN/RPS: International Society of Nephrology/ Renal Pathology Society
LN: Lupus nephritis
SLE: Systemic lupus erythematosus

## References

[1] Sada KE, Makino H (2009). Usefulness of ISN/RPS classification of lupus nephritis. J Korean Med Sci.

[2] Bajema IM, Wilhelmus S, Alpers CE, Bruijn JA, Colvin RB, Cook HT, D'Agati VD, Ferrario F, Haas M, Jennette JC (2018). Revision of the International Society of Nephrology/Renal Pathology Society classification for lupus nephritis: clarification of definitions, and modified National Institutes of Health activity and chronicity indices. Kidney Int.

[3] Mok CC, Lau CS (2003). Pathogenesis of systemic lupus erythematosus. J Clin Pathol.

[4] Schur PH (1995). Genetics of systemic lupus erythematosus. Lupus.

[5] Cervera R, Khamashta MA, Font J, Sebastiani GD, Gil A, Lavilla P, Doménech I, Aydintug AO, Jedryka-Góral A, de Ramón E (1993). Systemic lupus erythematosus: clinical and immunologic patterns of disease expression in a cohort of 1,000 patients. The European Working Party on Systemic Lupus Erythematosus. Medicine (Baltimore).

[6] Lech M, Anders HJ (2013). The pathogenesis of lupus nephritis. J Am Soc Nephrol.

[7] Bijl M, Reefman E, Horst G, Limburg PC, Kallenberg CG (2006). Reduced uptake of apoptotic cells by macrophages in systemic lupus erythematosus: correlates with decreased serum levels of complement. Ann Rheum Dis.

[8] Marto N, Bertolaccini ML, Calabuig E, Hughes GR, Khamashta MA (2005). Anti-C1q antibodies in nephritis: correlation between titres and renal disease activity and positive predictive value in systemic lupus erythematosus. Ann Rheum Dis.

[9] Trendelenburg M, Lopez-Trascasa M, Potlukova E, Moll S, Regenass S, Frémeaux-Bacchi V, Martinez-Ara J, Jancova E, Picazo ML, Honsova E (2006). High prevalence of anti-C1q antibodies in biopsy-proven active lupus nephritis. Nephrol Dial Transplant.

[10] Walport MJ, Davies KA, Botto M (1998). C1q and systemic lupus erythematosus. Immunobiology.

[11] Frangou E, Georgakis S, Bertsias G (2020). Update on the cellular and molecular aspects of lupus nephritis. Clin Immunol.

[12] Nishi H, Mayadas TN (2019). Neutrophils in lupus nephritis. Curr Opin Rheumatol.

[13] Schiffer L, Bethunaickan R, Ramanujam M, Huang W, Schiffer M, Tao H, Madaio MP, Bottinger EP, Davidson A (2008). Activated renal macrophages are markers of disease onset and disease remission in lupus nephritis. J Immunol.

[14] Zhang T, Wang M, Zhang J, Feng X, Liu Z, Cheng Z (2021). Association between tubulointerstitial CD8+T cells and renal prognosis in lupus nephritis. Int Immunopharmacol.

[15] Maroz N, Segal MS (2013). Lupus nephritis and end-stage kidney disease. Am J Med Sci.

[16] Xipell M, Lledó GM, Egan AC, Tamirou F, Del Castillo CS, Rovira J, Gómez-Puerta JA, García-Herrera A, Cervera R, Kronbichler A (2023). From systemic lupus erythematosus to lupus nephritis: The evolving road to targeted therapies. Autoimmun Rev.

[17] Juha M, Molnár A, Jakus Z, Ledó N (2023). NETosis: an emerging therapeutic target in renal diseases. Front Immunol.

[18] Bethunaickan R, Berthier CC, Ramanujam M, Sahu R, Zhang W, Sun Y, Bottinger EP, Ivashkiv L, Kretzler M, Davidson A (2011). A unique hybrid renal mononuclear phagocyte activation phenotype in murine systemic lupus erythematosus nephritis. J Immunol.

[19] Chen J, Cui L, Ouyang J, Wang J, Xu W (2022). Clinicopathological significance of tubulointerstitial CD68 macrophages in proliferative lupus nephritis. Clin Rheumatol.

[20] Jing C, Castro-Dopico T, Richoz N, Tuong ZK, Ferdinand JR, Lok LSC, Loudon KW, Banham GD, Mathews RJ, Cader Z (2020). Macrophage metabolic reprogramming presents a therapeutic target in lupus nephritis. Proc Natl Acad Sci U S A.

[21] Orme J, Mohan C (2012). Macrophage subpopulations in systemic lupus erythematosus. Discov Med.

[22] Mantovani A, Sica A, Sozzani S, Allavena P, Vecchi A, Locati M (2004). The chemokine system in diverse forms of macrophage activation and polarization. Trends Immunol.

[23] Ndisang JF, Mishra M (2013). The heme oxygenase system selectively suppresses the proinflammatory macrophage m1 phenotype and potentiates insulin signaling in spontaneously hypertensive rats. Am J Hypertens.

[24] Kulkarni O, Pawar RD, Purschke W, Eulberg D, Selve N, Buchner K, Ninichuk V, Segerer S, Vielhauer V, Klussmann S (2007). Spiegelmer inhibition of CCL2/MCP-1 ameliorates lupus nephritis in MRL-(Fas)lpr mice. J Am Soc Nephrol.

[25] Olmes G, Büttner-Herold M, Ferrazzi F, Distel L, Amann K, Daniel C (2016). CD163+ M2c-like macrophages predominate in renal biopsies from patients with lupus nephritis. Arthritis Res Ther.

[26] Bankhead P, Loughrey MB, Fernández JA, Dombrowski Y, McArt DG, Dunne PD, McQuaid S, Gray RT, Murray LJ, Coleman HG (2017). QuPath: Open source software for digital pathology image analysis. Sci Rep.

[27] Schwartz GJ, Muñoz A, Schneider MF, Mak RH, Kaskel F, Warady BA, Furth SL (2009). New equations to estimate GFR in children with CKD. J Am Soc Nephrol.

[28] Hill GS, Delahousse M, Nochy D, Rémy P, Mignon F, Méry JP, Bariéty J (2001). Predictive power of the second renal biopsy in lupus nephritis: significance of macrophages. Kidney Int.

[29] Kwant LE, Vegting Y, Tsang ASMWP, Kwakernaak AJ, Vogt L, Voskuyl AE, van Vollenhoven RF, de Winther MPJ, Bemelman FJ, Anders HJ (2022). Macrophages in lupus nephritis: exploring a potential new therapeutic avenue. Autoimmun Rev.

[30] Richoz N, Tuong ZK, Loudon KW, Patiño-Martínez E, Ferdinand JR, Portet A, Bashant KR, Thevenon E, Rucci F, Hoyler T (2022). Distinct pathogenic roles for resident and monocyte-derived macrophages in lupus nephritis. JCI Insight.

[31] Iwata Y, Boström EA, Menke J, Rabacal WA, Morel L, Wada T, Kelley VR (2012). Aberrant macrophages mediate defective kidney repair that triggers nephritis in lupus-susceptible mice. J Immunol.

[32] Boswell JM, Yui MA, Burt DW, Kelley VE (1988). Increased tumor necrosis factor and IL-1 beta gene expression in the kidneys of mice with lupus nephritis. J Immunol.

[33] Horuluoglu B, Bayik D, Kayraklioglu N, Goguet E, Kaplan MJ, Klinman DM (2019). PAM3 supports the generation of M2-like macrophages from lupus patient monocytes and improves disease outcome in murine lupus. J Autoimmun.

[34] Li F, Yang Y, Zhu X, Huang L, Xu J (2015). Macrophage polarization modulates development of systemic lupus erythematosus. Cell Physiol Biochem.

[35] Li J, Yu YF, Liu CH, Wang CM (2017). Significance of M2 macrophages in glomerulonephritis with crescents. Pathol Res Pract.

[36] Kishimoto D, Kirino Y, Tamura M, Takeno M, Kunishita Y, Takase-Minegishi K, Nakano H, Kato I, Nagahama K, Yoshimi R (2018). Dysregulated heme oxygenase-1(low) M2-like macrophages augment lupus nephritis via Bach1 induced by type I interferons. Arthritis Res Ther.

[37] Kaykı G, Orhan D, Gülhan B, Topaloğlu R, Akçören Z, Düzova A, Özaltın F, Özen S, Bilginer Y, Güçer Ş (2022). Glomerulonephritis with crescents in childhood; etiologies and significance of M2 macrophages. Turk J Pediatr.

[38] Zhao L, David MZ, Hyjek E, Chang A, Meehan SM (2015). M2 macrophage infiltrates in the early stages of ANCA-associated pauci-immune necrotizing GN. Clin J Am Soc Nephrol.

[39] Dias CB, Malafronte P, Lee J, Resende A, Jorge L, Pinheiro CC, Malheiros D, Woronik V (2017). Role of renal expression of CD68 in the long-term prognosis of proliferative lupus nephritis. J Nephrol.

[40] Zhang T, Li H, Vanarsa K, Gidley G, Mok CC, Petri M, Saxena R, Mohan C (2020). Association of urine sCD163 with proliferative lupus nephritis, fibrinoid necrosis, cellular crescents and intrarenal M2 macrophages. Front Immunol.

[41] Yang G, Guo N, Yin J, Wu J (2021). Elevated soluble CD163 predicts renal function deterioration in lupus nephritis: a cohort study in Eastern China. J Int Med Res.

[42] Anders HJ, Ryu M (2011). Renal microenvironments and macrophage phenotypes determine progression or resolution of renal inflammation and fibrosis. Kidney Int.

[43] Wynn TA, Chawla A, Pollard JW (2013). Macrophage biology in development, homeostasis and disease. Nature.

[44] Bao L, Osawe I, Puri T, Lambris JD, Haas M, Quigg RJ (2005). C5a promotes development of experimental lupus nephritis which can be blocked with a specific receptor antagonist. Eur J Immunol.

[45] Daniel C, Leppkes M, Munoz LE, Schley G, Schett G, Herrmann M (2019). Extracellular DNA traps in inflammation, injury and healing. Nat Rev Nephrol.

[46] Xue J, Schmidt SV, Sander J, Draffehn A, Krebs W, Quester I, De Nardo D, Gohel TD, Emde M, Schmidleithner L (2014). Transcriptome-based network analysis reveals a spectrum model of human macrophage activation. Immunity.

