# Supplementary material for: Macrophage subpopulations in pediatric patients with lupus nephritis and other inflammatory diseases affecting the kidney
Source: Arthritis Res Ther. 2024 Feb 8;26:46. doi: 10.1186/s13075-024-03281-1 (PMC10851514; doi:10.1186/s13075-024-03281-1)
Supplement: Supplementary file 1 — Additional file 1: Supplemental Table 1. Characteristics of adult LN cohort. Supplemental Table 2. Primary antibodies used for immunofluorescence microscopy. Supplemental Table 3. Secondary antibodies used for immunofluorescence microscopy (IF). [file 13075_2024_3281_MOESM1_ESM.docx]

**Supplemental Table 1: Characteristics of adult LN cohort.**

|  | **Lupus ISN-RPS classes** | | | |
| --- | --- | --- | --- | --- |
|  | **class II** | **class III** | **class IV** | **class V** |
| Number of patients [n] | 6 | 11 | 22 | 18 |
| Age of Patients [years] | 38.2±16.9 | 35.5±9.5 | 34.4±13.5 | 40.0±14.8 |
| Male [n] | 1 | 1 | 2 | 5 |
| Female [n] | 5 | 10 | 20 | 13 |
| Hypertensive patients [%] | 16.7 | 27.2 | 22.7 | 27.7 |
| Diabetic patients [%] | 0 | 0 | 0 | 11.1 |
| Serum creatinine [mg/dl] | 0.8±0.3 | 1.0±0.4 | 1.1±0.5 | 1.2±1.0 |
| Serum urea [mg/dl] | 23.2±16.5 | 33.0±12.5 | 43.5±18.7 | 27.6±27.0 |
| Glomerulosclerosis index [score 0-4] | 1.1±0.78 | 1.3±0.7 | 2.1±0.5 | 1.3±0.9 |

If variables are not expressed as a single value, data were shown as mean±SD.

**Supplemental Table 2: Primary antibodies used for immunofluorescence microscopy.**

| **No.** | **Antigen** | **Host** | **Ig-class** | **Dilution** | **Antigen retrieval** | **Supplier** |
| --- | --- | --- | --- | --- | --- | --- |
| 1 | CD3 | mc rat | n.d. | 1:50 | 2.5 min PC TRS 110°C | Bio-Rad GmbH, Feldkirchen, Germany |
| 2 | CD20 | mc mouse | IgG2a | 1:100 | 2.5 min PC TRS 110°C | Abcam plc, Cambridge, UK |
| 3 | CD68 | mc mouse | IgG3 | 1:100 | 2.5 min PC TRS 110°C | DAKO Deutschland GmbH, Hamburg, Germany |
| 4 | CD163 | Pc  goat | IgG1 | 1:100 | 2.5 min PC TRS 110°C | Novocastra, Leica Biosystems Newcastle Ltd; Newcastle, UK |
| 5 | CD206 | pc rabbit | IgG1 | 1:50 | 2.5 min PC TRS 110°C | Abnova, Jhongli City, Taiwan |
| 6 | Myeloperoxidase | pc rabbit | n.d. | 1:50 | 2.5 min PC TRS 110°C | Abcam plc, Cambridge, UK |

mc = monoclonal, pc= polyclonal, n.d.=not determined, PC=pressure cooker, TRS=Target retrieval solution pH6.

**Supplemental Table 3: Secondary antibodies used for immunofluorescence microscopy (IF).**

| **No.** | **Antigen** | **Host** | **Dilution** | **Application** | **Supplier** |
| --- | --- | --- | --- | --- | --- |
| 1 | anti-mouse IgG1 Cy3 | goat | 1:200 | IF | Dianova GmbH, Hamburg, Germany |
| 2 | anti-mouse IgG3 Alexa Fluor 488 | goat | 1:200 | IF | Dianova GmbH, Hamburg, Germany |
| 3 | anti-rabbit IgG Alexa Fluor 647 | donkey | 1:200 | IF | Invitrogen, Carlsbad, CA, USA |
| 4 | anti-rat IgG Alexa Fluor 647 | donkey | 1:200 | IF | Invitrogen, Carlsbad, CA, USA |

IF= Immunofluorescence
