# Supplementary material for: Macrophage subpopulations in pediatric patients with lupus nephritis and other inflammatory diseases affecting the kidney
Source: Arthritis Res Ther. 2024 Feb 8;26:46. doi: 10.1186/s13075-024-03281-1 (PMC10851514; doi:10.1186/s13075-024-03281-1)
Supplement: Supplementary file 2 — Additional file 2: Supplemental Figure 1. Association of glomerular inflammatory cells with macrophage subtypes. [file 13075_2024_3281_MOESM2_ESM.pdf]

A

	total CD68+	CD68+ CD206-	CD68+ CD163-	CD68+ CD206+	CD68+ CD163+	ratio CD206-/CD206+	ratio CD163-/CD163+
CD3+	0,376	0,313	0,41	0,398			0,343
MPO+	0,382	0,608	0,456			0,451	0,318

B

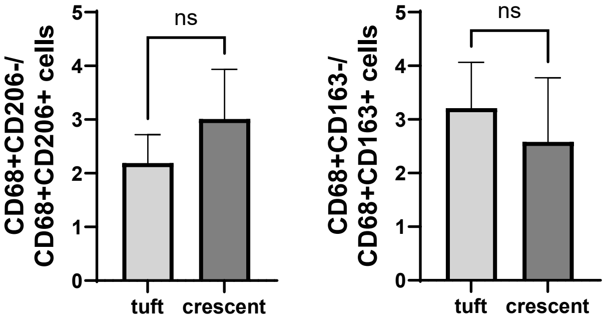

Suppl. Fig. 1

Supplemental Figure 1: **Association of glomerular inflammatory cells with macrophage subtypes.** A: Correlation of glomerular macrophage subtypes and M1-like/M2-like ratios with glomerular CD3+ and MPO+ cells, B: Comparison of CD68+CD206-/CD68+CD206+ and CD68+CD163-/CD68+CD163+ ratios in glomerular tuft and crescents.
